# Surgical approach for lens extraction from a crowded anterior segment

**DOI:** 10.5935/0004-2749.20210086

**Published:** 2021

**Authors:** Jagdeep Singh Gandhi

**Affiliations:** 1 Worcester Royal Eye Unit, Worcestershire Royal Hospital, Worcester WR5 1DD, United Kingdom

The anatomy of the anterior segment can pose difficulty for the surgeon. A parameter of interest is the depth of the anterior chamber. In the short eye, one expects a shallow anterior chamber. Yet the same feature can occur in the eye of typical axial length.

Cataract surgery in a crowded eye was discussed by Ozcura and Irgat^(1)^. To deepen the anterior chamber they used mannitol to shrink the vitreous. The authors noted the existence of a surgical solution: the retrolental vitreous can be removed. Surgeons of the anterior segment, to achieve this aim, have gone into the posterior segment. Passing a trocar via the pars plana, they have debulked the vitreous body. One group allowed the gel to exude and cut the flowing efflux on the eye surface^(2)^. Hence the vitreous can be shrunk medically or surgically to ease cataract extraction.

The specifics of anaesthesia are of importance. Vitreous pressure can rise after injection into the posterior subtenon space. General anaesthetic is thus desirable for a very shallow chamber (≤2mm). If general anaesthesia is not feasible the use of topical anaesthesia would be unwise. The residual choices are peribulbar block, or the method of advanced subconjunctival anaesthesia^(3)^. After these injections, ocular massage (or similar pressure) will soften the eyeball for surgery^(4)^.

At surgery the authors were taken with the billowing of the posterior capsule. They called the sight ‘floppy capsule’ in tandem with ‘floppy iris’. But capsular floppiness is not limited to the context of their report. A surgeon meets it episodically: after removing a big nucleus, or when operating in highly myopic or vitrectomised eyes. Also in cases with lax zonules, or in eyes with liquid vitreous. It is better to say that mannitol, which contracts the vitreous, is another cause of a ‘floppy capsule’.

If a capsule hinders intraocular surgery then so can constraints of space. In a shallow anterior chamber it is natural to steer clear of the corneal endothelium. Corneal decompensation is a concern. But as the plane of nucleo fractis moves backward the posterior capsule is under threat. When fracturing the nucleus the aim is to save the cornea and the capsule, and adequate space in the anterior segment is a precondition for safe surgery.

Commonly the bottle is raised to gain more intracameral space. Gravity pushes more fluid into the eye and expands the anterior chamber. But high fluid-pressure can soon hydrate the vitreous^(5)^. Longer surgery will, likewise, result in vitreous hydration. So the anterior vitreous bulges, and the posterior capsule swells massively forward^(6)^. At this stage, swirls of fluid can cause a chip of nucleus to strike the bulging capsule. The outcome is a rent in the membrane.

Surgical space can otherwise be enlarged by using re tentive viscoelastic. Options include Healon-5 or Healon-GV. With heavy viscoelastic, a stable matrix is formed in which microsurgery can be executed. The carving of a trench and cracking of the nucleus yields room for manoeuvre. Next dispersive viscoelastic. It is fed into the depth of the lens, and spills into the retronuclear plane. Impacted nucleus is dissected off the capsule. Next heavy viscoelastic. In a tiny space it safely moves about the nuclear fragments.

Exerting soft but strong force, heavy viscoelastic floats a freed wedge of lens. After adding viscoelastic, the cannula becomes a tool in the capsular bag. The surgery is now slowed down. There is no saline flow into the eye. But the anterior segment is formed. Intraocular calm allows us to mobilise the lens with control and comfort.

A barrier of heavy viscoelastic holds back the posterior capsule. The same viscoelastic shields the cornea, as portions of lens are emulsified bit by bit. Refills of heavy viscoelastic are injected during the ultrasonic phase. Opposite poles, the endothelium and the posterior capsule, are both guarded. Good use of viscoelastic again supports the posterior capsule, as fronds of cortex are peeled away. At the end, with lens implant in place, the viscoelastic is cleared with exactness. Lingering viscoelastic in the angle can cause a pressure rise and antipressure drugs may be needed after surgery.
